# Supplementary material for: Molecular detection of multidrug resistance pattern and associated gene mutations in M. tuberculosis isolates from newly diagnosed pulmonary tuberculosis patients in Addis Ababa, Ethiopia
Source: PLoS One. 2020 Aug 4;15(8):e0236054. doi: 10.1371/journal.pone.0236054 (PMC7402498; doi:10.1371/journal.pone.0236054)
Supplement: Attachment — Submitted filename: comments PONE-D-20-08561.docx [file pone.0236054.s002.docx]

**Comments to the authors:**

The present study deals with the characterization of drug-resistant patterns of newly identified and diagnosed TB cases in various parts of Addis Ababa, Ethiopia. Ethiopia is among the 30 high burden TB endemic region. Effective monitoring and management of TB cases are urgently required for the containment of the TB disease in high burden regions. The authors studied the resistant pattern in 190 cases of newly diagnosed TB. In the present study, the authors identified two risk factors that are associated with MDR-TB development i.e., chronic illness and HIV infection. Although, earlier reports mentioned higher INH mono-resistance or INH/RIF mono resistance prevalence in the regions of Ethiopia, one study has mentioned a similar pattern of mono resistance. The observed higher resistance cases of INH resistance maybe due to the prevalent use of INH as preventive therapy of TB in HIV infected cases in the region studied. Most of the resistance was due to the common mutations occurring in the INH as well as RIF gene. The authors also observed hetero-resistance cases to the INH, which might be due to the mixed isolates infection. The authors also find a low occurrence of MDR-TB cases in the study population, which has been reported to have higher percentage of MDR-TB cases. Overall, the manuscript cannot be considered for publication in present form and will require substantial improvement for consideration. The primary concern regarding the current manuscript is the sample size. The overall sample size is low and can affect the outcome of the study. The following queries may be considered before submitting the revision.

1. The authors mentioned that 190/230 patients were sputum smear as well as culture positive. The details of the method used in the drug sensitivity assay need inclusion in the manuscript.
2. The authors are advised to revise the information provided under the heading “**MDR-TB Associated Risk factors."** There appear some mistakes in the data entry.
3. Line no. 119: Author should mention the method of smear microscopy and reference should be quoted for the guidelines & procedures followed.
4. Line no. 129 Reference is missing.
5. Line no. 134- 135 “Microscopic examinations of culture colonies were done to select AFB positive isolates”. This statement needs to be rephrased for more clarity. How the isolates have been differentiated between MTB & NTMs? As per the WHO guidelines, smear microscopy for TB diagnosis is recommended by Auramine O staining (fluorescent microscopy) however the current study followed ZN method. Hence author should justify the use of ZN method for smear microscopy?
6. According to the WHO guidelines for TB diagnosis, sputum samples are needed to be collected for two consecutive days. In the present study, how the sputum samples have been collected? Whether these samples were from day 1 or day 2 or pooled? The author should clearly mention the collection of sputum samples from the patients enrolled.
7. Line no. 137 “AFB positive isolates” is not clear in this statement whether the direct examination of sputum smear or culture positive isolates confirmed by AFB smear microscopy were taken for molecular typing. The author has not mentioned number of AFB negative isolates throughout the study. The AFB negative isolates have to be investigated using PCR based deletion typing and molecular genetic assay and it must be included in the results. Author may provide supplementary file of data for more clarity.
8. Author should provide schematic diagram of study design for more clarity.
9. The data has not been analyzed by comparing the results with culture (gold standard method). Additionally, the present study has not done the phenotypic drug susceptibility testing which is must to validate the results of molecular drug resistance of isolates.
10. Author needs to reanalyze the data keeping culture as gold standard method.
11. In Table 5, rather than putting column “Types of mutation” author should mention the frequency of individual mutations. The table 3 is described in terms of monoresistance, any resistance and MDR, similarly in table 5 author should mention these terms in the column of “Resistance pattern”. The table 5 needs to be modified in more descriptive and informative way such as by mentioning the change in amino acid from wild type to mutant type with codon position.
12. Line no. 284-285 Author stated “……signaling there is also high and low-level INH resistance in the same isolates” on what basis the author has considered the low and high level of resistance?
13. Line no. 367 the author should correct the statement by replacing “ant-TB drugs” with “anti-TB drugs” and “cells” with “isolates”
14. ·The authors fail to explain the importance of the present study.
15. ·The study has very less number of samples to be compared to give any conclusion. The data provided in the manuscript is not efficiently analysed to conclude. Most of the claims of this manuscript are insubstantial and lacks quality for publishing in its present form.
16. Novelty is missing in the study.
17. The authors should revise the discussion part thoroughly in the manuscript which seems very weak to support the study.
18. References are missing at the relevant places.
19. The language of the manuscript need thorough revision for brevity.
