# Supplementary material for: Molecular detection of multidrug resistance pattern and associated gene mutations in M. tuberculosis isolates from newly diagnosed pulmonary tuberculosis patients in Addis Ababa, Ethiopia
Source: PLoS One. 2020 Aug 4;15(8):e0236054. doi: 10.1371/journal.pone.0236054 (PMC7402498; doi:10.1371/journal.pone.0236054)
Supplement: Attachment — Submitted filename: Response to Reviewers.docx [file pone.0236054.s003.docx]

Date 22/05/2020

Response to Reviewers

To: - PLOS ONE Reviewer,

Dear, Sir, Madam

We received the document which contains the reviewer's comments from the PLOS ONE with protocol PONE-D-20-08561 on Thursday, April 16/2020 at 9:27 AM. We would like to thank the reviewers for the attentive and thorough reading of this manuscript and for the thoughtful comments and constructive suggestions, which help to improve the quality of this manuscript. We try to jot down the reviewer's comments and respond to the comments accordingly.

**Comment:** The authors mentioned that 190/230 patients were sputum smear as well as culture positive. The details of the method used in the drug sensitivity assay need inclusion in the manuscript.

**Response:** Comment accepted and the detailed method used in the drug sensitivity assay is added to the manuscript (pp 8)

**Comment:** The authors are advised to revise the information provided under the heading “**MDR-TB Associated Risk factors."** There appear some mistakes in the data entry.

**Response:** Comment accepted and corrected accordingly (pp 12)

**Comment:** Line no. 119: The author should mention the method of smear microscopy and reference should be quoted for the guidelines & procedures followed.

**Response:** Comment accepted and corrected accordingly (pp 6)

**Comments:** Line no. 129 Reference is missing.

**Response:** Comment accepted and corrected accordingly (pp 7)

**Comment:** Line no. 134- 135 “Microscopic examinations of culture colonies were done to select AFB positive isolates”. This statement needs to be rephrased for more clarity. How the isolates have been differentiated between MTB & NTMs? As per the WHO guidelines, smear microscopy for TB diagnosis is recommended by Auramine O staining (fluorescent microscopy) however the current study followed the ZN method. Hence the author should justify the use of the ZN method for smear microscopy?

**Response:** Comment accepted and the statement is rephrased to provide more information. The typing was done using the RD9 deletion typing to differentiate MTBC from NTMs. Since the study was conducted in the research center we have used the ZN method because ZN is a gold standard and auramine is for program efficiency (pp 6 - 7).

**Comment:** According to the WHO guidelines for TB diagnosis, sputum samples are needed to be collected for two consecutive days. In the present study, how the sputum samples have been collected? Whether these samples were from day 1 or day 2 or pooled? The author should clearly mention the collection of sputum samples from the patients enrolled.

**Response:** Comment accepted and the procedure is further elaborated to avoid confusion. Accordingly, front-loading is well accepted, WHO has modified case definitions; in any case, the study can have its method and two samples are fine with >97% sensitivity (pp6).

**Comment:** Line no. 137 “AFB positive isolates” is not clear in this statement whether the direct examination of sputum smear or culture-positive isolates confirmed by AFB smear microscopy were taken for molecular typing. The author has not mentioned the number of AFB negative isolates throughout the study. The AFB negative isolates have to be investigated using PCR based deletion typing and molecular genetic assay and it must be included in the results. The author may provide a supplementary file of data for more clarity.

**Response:** Comment accepted and the statement has been corrected. In this study, there were no AFB negative isolates because all were smear-positive by entry criterion (pp6 - 7).

**Comment:** The author should provide a schematic diagram of the study design for more clarity.

**Response:** Comment accepted and corrected accordingly (Fig 1 supplement file)

**Comment:** The data has not been analyzed by comparing the results with culture (gold standard method). Additionally, the present study has not done the phenotypic drug susceptibility testing which is a must to validate the results of molecular drug resistance of isolates.

**Response:** Comment accepted and clearly stated in the limitation part of this study

**Comment:** The author needs to reanalyze the data-keeping culture as a gold standard method.

**Response:** The study is not a methods comparison. In any case, all were culture positive and none were PCR negative.

**Comment:** In Table 5, rather than putting column “Types of mutation” the author should mention the frequency of individual mutations. Table 3 is described in terms of monoresistance, any resistance, and MDR, similarly, in table 5 author should mention these terms in the column of “Resistance pattern”. Table 5 needs to be modified in a more descriptive and informative way such as by mentioning the change in amino acid from wild type to mutant type with codon position.

**Response:** Comment accepted and corrected accordingly and the tables are completely modified by new formats Table 2 (pp12) and table 4 (pp 14 )

**Comment:** Line no. 284-285 The Author stated, “……signaling there is also high and low-level INH resistance in the same isolates” on what basis the author has considered the low and high level of resistance?

**Response:** Comment accepted and corrected accordingly (pp14)

**Comment:** Line no. 367 the author should correct the statement by replacing “ant-TB drugs” with “anti-TB drugs” and “cells” with “isolates”

**Response:** Comment accepted and corrected accordingly (pp 18)

**Comment:** The authors fail to explain the importance of the present study.

**Response:** Comment accepted and corrected accordingly (pp 4 – 5)

**Comment:** The study has a very less number of samples to be compared to give any conclusion. The data provided in the manuscript is not efficiently analyzed to conclude. Most of the claims of this manuscript are insubstantial and lack quality for publishing in its present form.

**Response:** Comment accepted and corrected accordingly. The sample size determination for the study primarily focuses on the required number of smear-positive pulmonary TB cases. The study was included all presumptive pulmonary tuberculosis cases during the study period (260 cases) from the selected 20 health facility, these health facilities were purposely selected because more TB patients were seen in these health facilities during the study period.

**Comments:** Novelty is missing in the study.

**Response:** The novelty of this study lies in the update it gives of the status in Addis Ababa in 2017 demonstrating that there is no rise in RR or MDR-TB.

**Comment:** The authors should revise the discussion part thoroughly in the manuscript which seems very weak to support the study.

**Response:** Comment accepted and corrected accordingly (pp 16 )

**Comment:** References are missing in the relevant places.

**Response:** Comment accepted and corrected accordingly

**Comment:** The language of the manuscript need a thorough revision for brevity.

**Response:** Comment accepted and corrected accordingly
